# An in vitro study of an *Artocarpus heterophyllus* substance as a hepatitis C antiviral and its combination with current anti-HCV drugs

**DOI:** 10.1186/s12906-021-03408-w

**Published:** 2021-10-12

**Authors:** Adita Ayu Permanasari, Chie Aoki-Utsubo, Tutik Sri Wahyuni, Lidya Tumewu, Myrna Adianti, Aty Widyawaruyanti, Hak Hotta, Achmad Fuad Hafid

**Affiliations:** 1 Institute of Tropical Disease, Universitas Airlangga, Surabaya, 60115 Indonesia; 2 Department of Public Health, Kobe University Graduate School of Health Sciences, 7-10-2, Tomogaoka, Suma-ku, Kobe, 654-0142 Japan; 3 Department of Pharmaceutical Sciences, Faculty of Pharmacy, Universitas Airlangga, Surabaya, 60115 Indonesia; 4 Department of Health, Study Program Traditional Medicine, Vocational Faculty, Universitas Airlangga, Surabaya, Indonesia; 5 Faculty of Clinical Nutrition and Dietetics, Konan Women’s University, 6-2-23, Morikita-machi, Higashida-ku, Kobe, 658-0001 Japan

**Keywords:** Hepatitis, *Artocarpus heterophyllus*, Medicine, Infectious Disease

## Abstract

**Background:**

Current therapy of chronic hepatitis C virus (HCV) with direct-acting antivirals (DAAs) has dramatically improved the sustained virologic response (SVR) of affected patients; however, treatment with DAAs remains expensive, and drug-resistant HCV variants remain a threat. As a result, there is still a need to continue to develop affordable and effective drugs for the treatment of HCV. Previously, we have demonstrated that a crude extract from *Artocarpus heterophyllus* leaves is a potential anti-HCV candidate. In this study, we have further purified this crude extract, examined which sub-fraction possesses the highest antiviral activity, and then explored its efficacy at different HCV life cycle stages. We also assessed synergistic antiviral effects between the *A. heterophyllus* extract and commercially available anti-HCV drugs.

**Methods:**

We used vacuum liquid chromatography (VLC) and high-performance liquid chromatography (HPLC) to fractionate a dichloromethane extract of *A. heterophyllus* leaves. We then examined the anti-HCV activity of the fractions using HCV genotype 2a, JFH1a; the antiviral mode of action was determined by exploring adding the treatments at different times. We examined the antiviral effects on the viral entry stage through a virucidal activity test, viral adsorption examination, and pretreatment of cells with the drug. The effects on the post-viral entry stage were determined by the levels of HCV protein expression and HCV RNA expression in infected cells.

**Results:**

Through activity guided purification, we identified the sub-fraction FR3T3 as possessing the most robust anti-HCV activity with an IC_50_ value of 4.7 ± 1.0 μg/mL. Mode-of-action analysis revealed that FR3T3 inhibited post-viral entry stages such as HCV NS3 protein expression and HCV RNA replication with marginal effects on the viral entry stage. Thin-layer Chromatography (TLC) indicated that FR3T3 contained terpenoids and chlorophyll-related compounds. We also found a synergistic antiviral activity when the DCM extract of *A. heterohyllus* was used in combination therapy with commercial anti-HCV drugs; Ribavirin, Simeprevir, Cyclosporin A.

**Conclusions:**

The extract of *A. heterophyllus* and its sub-fraction, FR3T3, presented here have anti-HCV activities and could be candidate drugs for add-on-therapy for treatment of chronic HCV infections.

## Background

The hepatitis C virus (HCV) is a positive-sense single-stranded RNA virus of the Flaviviridae family. The HCV genome is 9.6 kb in length and encodes three structural proteins (Core, E1, and E2) and seven non-structural proteins (p7, NS2, NS3, NS4A, NS4B, NS5A, NS5B). The structural proteins El and E2 are responsible for binding the virus to the receptor(s) on the host cell’s surface [1]. The non-structural proteins play an essential role in RNA replication, virus assembly, and virus release [2]. The HCV life cycle is mainly divided into seven steps: (1) virus attachment, (2) entry, (3) uncoating, (4) translation, (5) RNA genome replication, (6) assembly and maturation, and (7) virion release [3, 4].

HCV infection is a significant global health burden; it is estimated that 71 million people globally have a chronic HCV infection [5]. HCV causes both acute and chronic hepatitis. Patients with a chronic HCV infection are at a high risk of developing cirrhosis and hepatocellular carcinoma (HCC). Approximately 400 thousand people die every year due to HCV-related complications [6]. HCV strains are classified into seven genotypes (1 to 7) which are distributed worldwide [7]. Direct-acting antivirals (DAAs) are an effective therapy for HCV that target viral proteins such as NS3/NS4A protease, the NS5A protein, and NS5B polymerase, which are involved in viral replication. There are two generations of NS3/4A protease inhibitors: Boceprevir and Telaprevir are considered 1st generation treatments and Faldaprevir, Asunaprevir, Vaniprevir, Paritaprevir, Grazoprevir, Sovaprevir, and Simeprevir are considered 2nd generation. There are also two generations of NS5A protein inhibitors: Daclastavir, Ledipasvir, and Ombitasvir are considered 1st generation and Elbasvir, Velpatasvir, Odalasvir, are considered 2nd generation. There are two groups of NS5B polymerase inhibitors, another class of DAAs: Nucleoside Polymerase Inhibitor’s (NPIs) such as Sofosbuvir, and Non-NPIs (NNPIs) such as Dasabuvir [8].

Oral DAA treatment achieves a very high (> 90%) sustained virological response (SVR) rate in patients with all genotypes of HCV. However, their expense prevents them from being widely used, particularly in low-income countries. As a result, access is limited to HCV treatment for many in need of it. Furthermore, the emergence of HCV strains that are resistant to DAAs is increasing in prevalence [9–12]. Therefore, there is still a requirement to develop safe and cost-effective alternative anti-HCV agents.

Natural products derived from plants have been used as healing agents for thousands of years. Plants produce a wide variety of secondary metabolites such as flavonoids, terpenoids, lignans, sulphides, polyphenolics, coumarins, saponins, furyl compounds, alkaloids, polyines, thiophenes, proteins, and peptides. Many of these plant chemicals have been reported to possess numerous bioactivities, including antiviral activity. Therefore, medicinal plants are an attractive source for screening antiviral drugs and may lead to the development of new anti-HCV agents [13, 14].

*Artocarpus* spp. are widely cultivated in tropical countries, including Indonesia, and have been used to treat a range of conditions such as skin diseases, diarrhea, and inflammation [15, 16]. *Artocarpus heterophyllus* has previously been reported to be effective against Herpes Simplex Virus (HSV), Human Immunodeficiency Virus (HIV), and Varicella-Zoster Virus (VZV) [17–20]. In our previous research, we found that *A. heterophyllus* leaves exhibit anti-HCV activity. In particular, a dichloromethane extract showed the most potent anti-HCV activity with an IC_50_ value of 1.5 μg/mL [21]. In this study, we fractionate this dichloromethane extract from *A. heterophyllus* leaves and analyze its anti-HCV activity mechanism of action. Finally, we determine the effectiveness of the dichloromethane (DCM) extract of *A. heterophyllus* with various current HCV drugs as a treatment for HCV infections.

## Methods

### General materials

Silica gel 60 GF_254_ (Merck) was used for vacuum liquid chromatography. Thin-layer Chromatography (TLC) was carried out using silica gel 60 F_254_ and RP-18 F_254_ plates (Merck). High-performance liquid chromatography (HPLC) was conducted using a Shimadzu system equipped with a LC-6 AD pump and a Diode Array Detector (SPD-M20A), as well as a Zorbax Eclipse XDB-C18 column (9.4 × 250 mm, 5 μm particle size, Agilent); mobile phase acetonitrile – water (9:1 v/v); flowrate 1 mL/min, injection volume 500 μL, wavelength 254 nm and 365 nm. HPLC solvents were purchased from Merck.

### Crude extract preparation, extraction, and fractionation

The leaves of *Artocarpus heterophyllus* Lam. were obtained from Purwodadi Botanical Garden, Indonesian Institute of Sciences, East Java, Indonesia and received approval for sampling according to regulations Peraturan LIPI nomor 26 tahun 2019. The species was verified by Mr. Matrani as an expert botanist of Purwodadi Botanical Garden, Indonesian Institute of Science, East Java, Indonesia. The voucher speciment has been deposited in material room at Institute of Tropical Disease, Universitas Airlangga by code AH01.

The Artocarpus leaves were extracted using n-hexane, which yielded a crude n-hexane extract (10.8 g). Meanwhile, the residue from n-hexane extract was further processed using dichloromethane (DCM) to generate 32.8 g of DCM extract. The DCM extract was further purified by using bioactivity guided fractionation. The DCM extract was applied to a silica gel vacuum column and eluted in a 25% gradient of *n*-hexane-dichloromethane (100:0 to 0:100) and a 15% gradient of dichloromethane-MeOH (100:0 to 90:10). This approach yielded four fractions (FR1 ~ FR4) which were identified based on their TLC profiles. Fraction FR3 (2.4 g) was further partitioned using HPLC (RP-18) and an elution gradient of ACN-H_2_O (9:1) which yielded a further seven sub-fractions (FR3T1 ~ FR3T7). All extracts, fractions, and sub-fractions were dissolved in dimethyl sulfoxide (DMSO) at a concentration of 100 mg/mL and then stored at − 30 °C before used for anti-HCV assay.

### Cells and viruses

A clone from a human hepatoma derived cell line, Huh7it-1 cells [22, 23], were cultured in Dulbecco’s Modified Eagle Medium (GIBCO Invitrogen, Carlsbad, CS, USA) supplemented with 10% Fetal Bovine Serum (Biowest, Nualle, France), 0.15 mg/mL Kanamycin (Sigma–Aldrich, St. Louis, MO, USA), and non-essential amino acids (GIBCO-Invitrogen) in 5% CO_2_ at 37 °C. A cell culture-adapted HCV variant was propagated as described previously [21, 22, 24]. In brief, a virus culture was created by collecting the supernatant from a Huh7it-1 cell culture infected by HCV JFH1. The supernatant was collected on the third to fifth day after infection and then concentrated using an Amicon filter and stored at − 80 °C.

### Virus titration and immunostaining

Virus titration and immunostaining were performed as described previously [21, 22, 24]. HCV JFH1 was cultivated in Huh7it-1 cells, which were then visualized through immunostaining. The culture supernatant from anti-HCV assay was dilluted 20-fold with medium then inoculated onto cell. Four hours after virus absorption, the remaining virus was removed, and cells were incubated with a medium containing 0.4% methylcellulose (Sigma-Aldrich) for 40 h. The immunostaining was performed to determine focus formation assay through the infectious foci. Firstly, Cells were fixed using 10% formaldehyde (200 μl per well) then washed 3x with PBS 200 μl/well. To permeable cell membrane, triton X 0.5% (100 μl per well) was added and the cells were incubated for 10 min. HCV infected patient serum was used to stain HCV antigen-positive cells by combining them at a 1:200 ratio with a solution of BlockAce (2%), BSA (1%), PBS and incubated for 1 h. We continued by adding a HRP-goat anti-human Ig antibody (MBL, tokyo, Japan) at a ratio of 1:400 under the same conditions. The enzymatis reaction was identified through reacting HRP and metal enhanced DAB substrate (ThermoFisher ScientificInc., Rockford, IL,USA) which resulted brown color for infected cells. The infectious foci were counted under an inverted microscope.

### Antiviral activity assay

Antiviral activity tests were performed as described previously [21, 22, 24]. In brief, Huh7it-1 cells (5.4 × 10^4^) were challenged with HCV at a multiplication of infection (MOI) of 0.1 in the presence of different concentrations of fractions or sub-fractions. Two hours after virus adsorption, the cells were rinsed with the medium and were further incubated in the medium for 46 h at 37 °C incubator.

### Time addition experiment

To determine the inhibition mechanism of the most active sub-fraction against HCV, a time addition experiment was carried out. Entry stage inhibition was tested using HCV JFH1 (MOI 0.1) and medium containing sample cells for 2 h and then incubated for 46 h with added medium without sample. Post entry step inhibition was tested by inoculating cells with HCV, incubating for 2 h, and then adding the sub-fraction and incubating for a further 46 h. Both stage inhibition was performed by added medium containing sample at 2 h and 46 h incubation. After 48 h post-infection (PI) culture supernatants were collected for virus titration. The 50% inhibitory effect (IC50) was calculated by using the SPSS probit analysis.

### MTT assay

The cytotoxicity of the samples to the cells was assessed using a 3-(4,5-Dimethylthiazol-2-Yl)-2,5-Diphenyltetrazolium Bromide (MTT) assay as described previously [21, 25]. Huh7it-1 cells (2.4 × 10^4^) placed in a 96 well plate were combined with sample at various concentrations and incubated for 48 h. After incubation, the medium was discarded and 150 μL of medium containing MTT (15 μL) was added and incubated for a further 4 h. Then 100 μL of DMSO was added to dissolve the precipitate that formed from the MTT reaction. The absorbance was measured at 560 nm and 750 nm wavelengths using the GloMax Microplate Multidetection Reader (Promega). Measurement results compared with a control. The resulting CC_50_ value was analyzed using SPSS analysis.

### Virucidal activity assay

A virucidal activity test was performed as described previously [21, 23]. In brief, a HCV JFH1 1 × 10^6^ FFU/mL as much as 75 mL was mixed with the sample and incubated for 2 h at 37 °C. Cells were then inoculated with 1250 dilutions and incubated for a further 4 h. After that the virus inoculum was removed, MC-DMEM medium was added to the cells and incubated for a further 40 h. Visualization of infected cell colonies was carried out by staining using DAB.

### Effect of host expression assay

Huh7it-1 cells (5.4 × 10^4^) were pretreated with a sub-fraction from *A. heterophyllus* for 2 h at 37 °C. The cells were then challenged with HCV (MOI of 0.1) for 2 h. The culture supernatant at 46 hpi was collected for virus titration.

### Immunoblotting

HCV infected cells were lysed in a RIPA buffer, and the protein concentrations were determined using a BCA assay kit (Thermo Fisher Scientific). Equal amounts of proteins were separated using SDS polyacrylamide gel electrophoresis and transferred onto a polyvinylidene difluoride (PVDF) membrane (Millipore, Bed-ford, MA, USA). The membranes were first probed with primary antibodies: a HCV NS3 mouse monoclonal antibody (clone H23; Abcam, Cambridge, MA, USA) and a β-actin antibody (MBL, Nagoya, Japan) followed by a secondary antibody, HRP-conjugated goat anti-mouse immunoglobulin (MBL) [21, 25]. Target proteins were visualized using an enhanced chemiluminescence detection system (Biorad; GE Healthcare, UK).

### Quantitative reverse transcription-polymerase chain reaction (qRT-PCR)

Extraction of total Ribonucleic Acid (RNA), cDNA preparation, and gene expression quantification by qPCR was performed as described previously [21, 26, 27]. Briefly, RNA was extracted from cells using Trizol. One microgram of total RNA was reverse transcribed using a Reverse Transcription System (Toyobo) using random primers. Real-time quantitative PCR analysis was performed using SYBR Premix Ex Taq (TaKaRa, Kyoto, Japan) on a MicroAmp 96 well plate. The primers used to amplify the region were NS3 5′-CTTTGACTCCGTGATCGACT-3′(sense) and 5′-CCCTGTCTTCCTCTACCTG-3′(antisense).

### Combination treatment experiments

The IC_50_ values of commercial antiviral drugs: Telaprevir (Tl) (Adooq Bioscience, Irvine, CA); Simaprevir (Sm) (Toronto Research Chemical, Canada); Ribavirin (Rb) (Sigma Aldrich, MO), and Cyclosporin A (Cy) (WAKO pure chemical, Japan) were determined using SPSS. Combination treatment experiments were conducted at 4x, 2x, 1x, 0.5x, and 0.25x of IC_50_ for each drug. Huh7it-1 cells were challenged with HCV in the presence of a mixture of *A. heterophyllus* extract and commercial drugs at the indicated concentrations. Compusyn software was used to determine the combination index value (CI). These were defined as: synergism effect: CI < 1, addictive effect: CI = 1, and antagonism effect: CI > 1 [22, 28].

## Results

### Fractionation of the *A. heterophyllus* dichloromethane extract

**Fig. 1:**
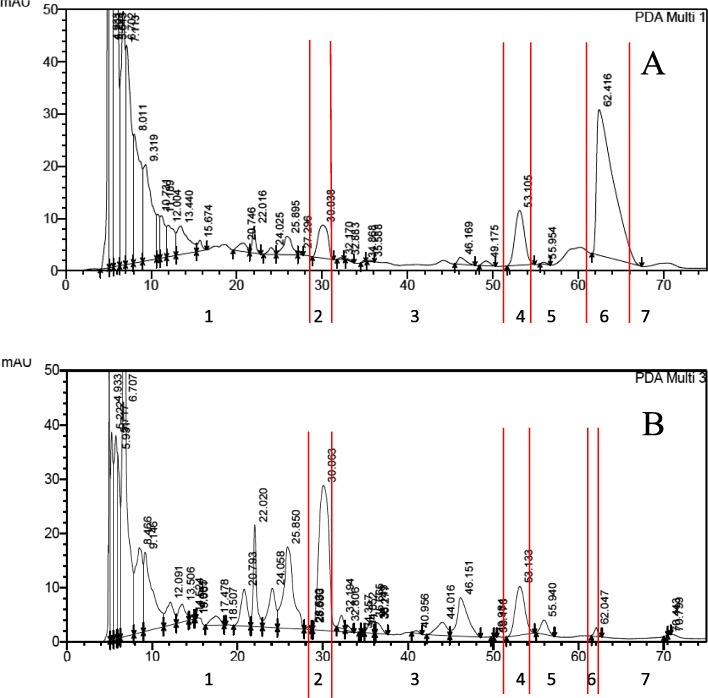
The FR3 sub-fractionation chromatogram using High Performance Liquid Chromatography (HPLC) at λ 254 nm and 365 nm. 1). Sub-fraction 1 (FR3T1), 2). Sub-fraction 2 (FR3T2), 3). Sub-fraction 3 (FR3T3), 4). Sub-fraction 4 (FR3T4), 5). Sub-fraction 5 (FR3T5), 6). Sub-fraction 6 (FR3T6), 7). Sub-fraction 7 (FR3T7)

Four fractions (FR1-FR4) were obtained from the dichloromethane extract of *A. heterophyllus* using Vacuum Liquid Chromatography (VLC). Bioassay results demonstrated that FR3 and FR4 exhibited strong anti-HCV activities and therefore was subjected to further separation by preparative HPLC. This approach resulted in the isolation of seven sub-fractions (FR3T1-FR3T7) (Fig. 1).

In total, four fractions and seven sub-fractions were isolated from the *A. heterophyllus* dichloromethane extract. FR3T6 was the most abundant sub-fraction (11.9 mg; Table 1), and FR3T2 was the least abundant sub-fraction (0.3 mg; Table 1).

**Table 1:** Weight and yield of fractions and sub-fractions of *A. heterophyllus* dichloromethane extract

Sample	Sample name	Sample code	Weight (mg)	Yield (%)
Extract	DCM Extract	–	4000.0	–
Fraction	Fraction 1	FR1	49.0	1.225
	Fraction 2	FR2	577.0	14.425
	Fraction 3	FR3	2591.0	64.775
	Fraction 4	FR4	70.0	1.75
Sub-fraction	Fraction 3 T1	FR3T1	20	10
	Fraction 3 T2	FR3T2	0.3	0.15
	Fraction 3 T3	FR3T3	8.5	4.25
	Fraction 3 T4	FR3T4	1.3	0.65
	Fraction 3 T5	FR3T5	2.2	1.1
	Fraction 3 T6	FR3T6	11.9	5.95
	Fraction 3 T7	FR3T7	3.8	1.9

### The anti-HCV activity of *A. heterohpyllus* sub-fractions

We found five sub-fractions (FR3T1, FR3T2, FR3T3, FR3T5 and FR3T7) possessed strong anti-HCV activities (IC_50_ values of < 10 μg/mL). Sub-fraction FR3T4 and FR3T6 did not show any antiviral activity at the tested concentration. Cytotoxicity results showed that FR3T3 was the least toxic in Huh7it-1 cells (CC_50_ > 100 μg/mL) among five active subfractions. Sub-fractions FR3T1, FR3T5, and FR3T7 exhibited strong cytotoxic effects on Huh7it-1 cells (CC_50_ < 60 μg/mL) (Table 2). Based on these results, we focused on sub-fraction FR3T3 in further experiments. This was principally to elucidate the mechanism behind the anti-HCV effects demonstrated by this sub-fraction.

**Table 2:** IC_50_, CC_50_ and SI values of fractions and subfractions of *A. heterophyllus* leaves dichloromethane extracts

Sample		IC_**50**_ (μg/mL)	CC_**50**_ (μg/mL)	Selectivity Index
Fraction	FR1	> 100	> 1000	> 10
	FR2	48.27 ± 8.82	> 1000 (1008.27 ± 28.23)	> 20.72
	FR3	3.79 ± 2.35	> 100 (193.77 ± 9.40)	> 26.39
	FR4	4.60 ± 1.46	> 100 (191.28 ± 0.02)	> 21.76
Subfraction	FR3T1	6.15 ± 0.60	> 50 (94.28 ± 8.44)	> 8.13
	FR3T2	< 3.12	> 25 (31.90 ± 5.34)	> 8.01
	FR3T3	4.69 ± 0.95	> 100 (130.14 ± 27.92)	> 21.32
	FR3T4	42.03 ± 2.92	> 200 (251.21 ± 1.75)	> 4.76
	FR3T5	6.84 ± 1.15	> 25 (38.76 ± 0.07)	> 3.65
	FR3T6	30.42 ± 1.23	> 400 (417.38 ± 77.23)	> 13.15
	FR3T7	2.39 ± 0.34	> 12.5 (16.16 ± 9.75)	> 5.23

The experiment was performed in triplicate

Firstly, we examined the effect of FR3T3 on the viral entry and post-entry stage by conducting time-of-addition experiments. Huh7it-1 cells were infected with HCV in the presence or absence of FR3T3 at different points in time. The entry-stage inhibition was determined by FR3T3 addition before viral infection; while the post-entry stage inhibition was determined by FR3T3 addition after viral infection. We also investigated the antiviral impact on both stages, simultaneously adding FR3T3 both before and after virus infection. We found that a 10 μg/mL treatment of FR3T3 at the entry or post-entry stages inhibited HCV by 33.9 and 64%, respectively. While the treatment at both stages inhibited HCV by 83% (Table 3). Furthermore, increasing the treatment dose of FR3T3 to 20 μg/mL, increased the suppression of HCV activity to 61.7% at the viral entry stage, 83.9% at the post-entry stage, and 93.4% when the treatment was applied at both stages simultaneously (Table 3).

**Table 3:** Time-of-addition experiment of FR3T3

No	Sample	Entry inhibition (%)	Post-entry inhibition (%)	Entry and post-entry inhibition (%)
1	FR3T3 (10 μg/mL)	33.86 ± 2.19	64.04 ± 3.06	83.07 ± 4.00
2	FR3T3 (20 μg/mL)	61.68 ± 0.10	83.86 ± 2.58	93.44 ± 5.29

The experiment was performed in triplicate

**Fig. 2:**
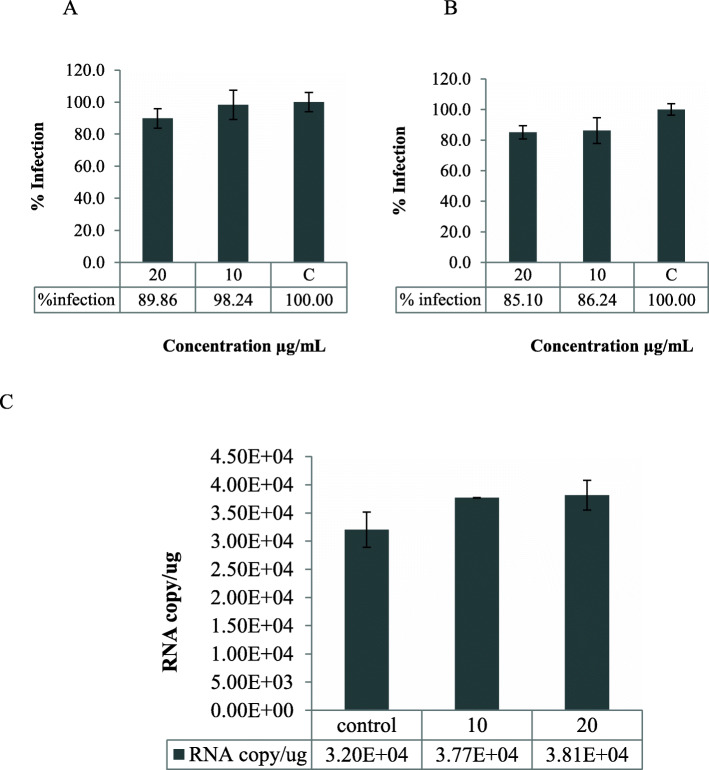
The results of the mode of action assays from the entry stage. **A** The percentage of HCV infection in the virucidal activity assay of the FR3T3 sub-fraction, **B** the percentage of HCV infection in the host cell expression activity assay, **C** Number of copies of RNA from the VHC absorption test on FR3T3 sub-fraction treated Huh7it-1 cells

Next, we performed three experiments to determine the mode of action at the entry stage. Firstly, through a virucidal activity test we examined how pretreatment of cells with FR3T3 influenced HCV infectivity and HCV adsorption. We found that FR3T3 at a dose of 20 μg/mL reduces HCV virion infectivity by 10.1%, compared to an untreated control (Fig. 2A). Pretreatment of cells with FR3T3 inhibited HCV infection by 14.9% compared to the untreated control (Fig. 2B); yet, FR3T3 did not block HCV adsorption to the surface of Huh7it-1 cells (Fig. 2C). These results suggested that FR3T3 exerts anti-HCV activity through both a direct virucidal effect and stimulating a host-related factor that influences viral entry; however, this antiviral impact at viral entry stage is relatively minor.

**Fig. 3:**
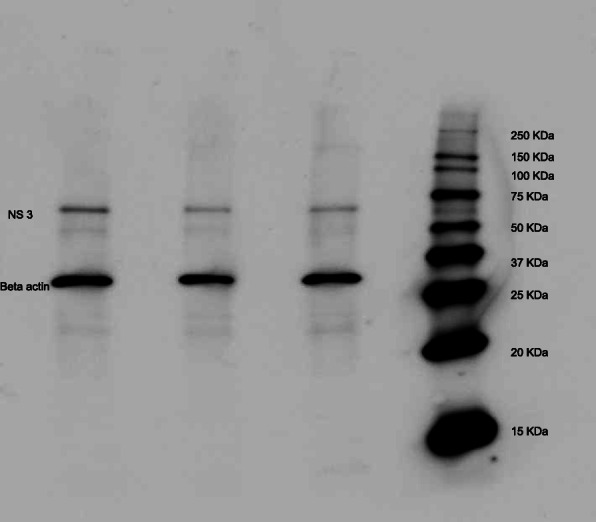
The expression of HCV NS3 proteins after the treatment of cells post-viral entry

**Fig. 4:**
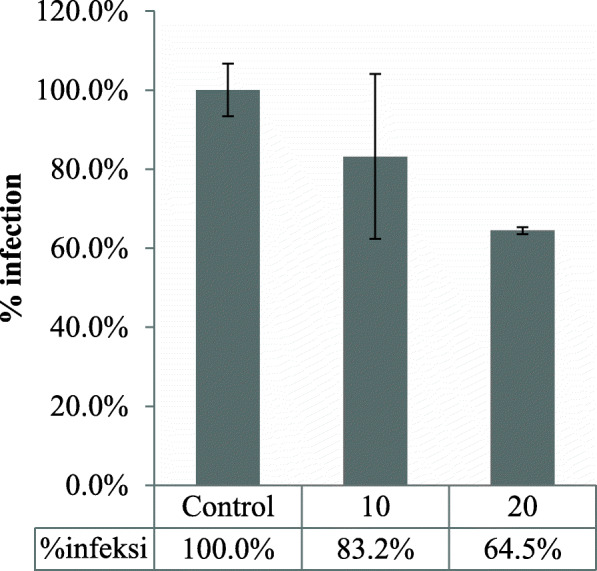
The percentage of infection from HCV RNA replication after the administration of FR3T3 at a concentration of 10 and 20 μg/mL

Next, we assessed the effect of FR3T3 at the post-viral entry stage. The FR3T3-containing medium was added to the cell culture after HCV infection, and the infected cells were incubated for 46 h. The infected cells were analyzed for the levels of NS3 protein expression and HCV RNA replication in the cells. The immunoblotting results showed FR3T3 decreased the expression of NS3 protein compared with the untreated control (Fig. 3). Similarly, we observed inhibition of HCV RNA replication in the FR3T3-treated cells. A 20 μg/mL dose of FR3T3 reduced HCV RNA levels in treated cells by 35.5% compared to the untreated control (Fig. 4). These results suggested that FR3T3 suppresses HCV replication after HCV entry.

### Chromatogram profiles of the DCM extract and fractions by TLC and LCMS

**Fig. 5:**
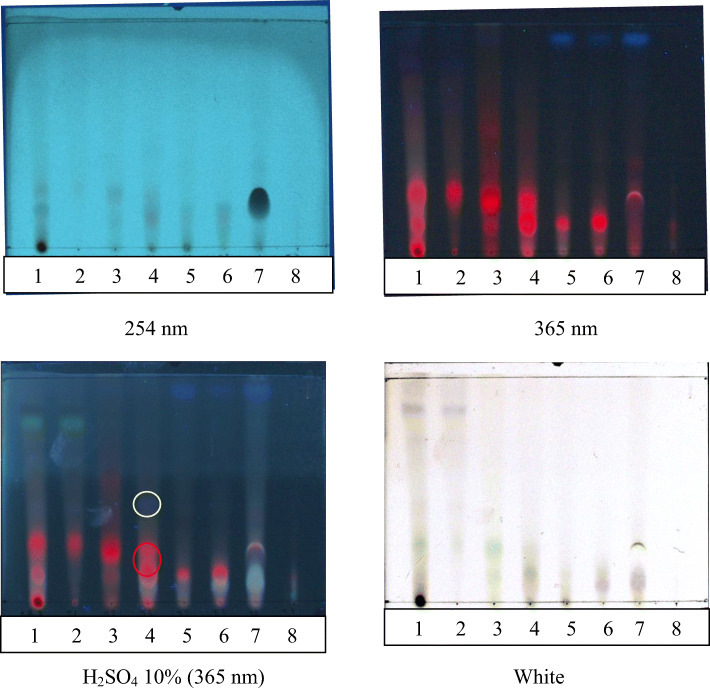
The TLC pattern of sub-fraction 1–7 of FR3T. RP-18 TLC was used as stationary phase and methanol:water (95:5, v/v) as a mobile phase. (1) *A. heterophyllus* dichloromethane extract, (2) FR3T1, (3) FR3T2, (4) FR3T3, (5) FR3T4 (6) FR3T5, (7) FR3T6, (8) FR3T7 sub-fraction. Detection under **A** UV 254 nm, **B** UV 365 nm, **C** sprayed with 10% sulfuric acid and heated at 105 °C for 5 min then observed under UV 365 nm **D** observed under white lamp

To elucidate the derivates that were responsible for anti-HCV activity in the FR3T3 sub-fraction, we conducted TLC analysis. Dark spots were observed under UV at 254 nm (Fig. 5A) and red spots were observed under UV at 365 nm (Fig. 5B and C). A green and purple spot was found after the resulting profile was sprayed with 10% sulfuric acid (Fig. 5D) which indicated that FR3T3 contains terpenoids and chlorophyll as major compounds.

**Fig. 6:**
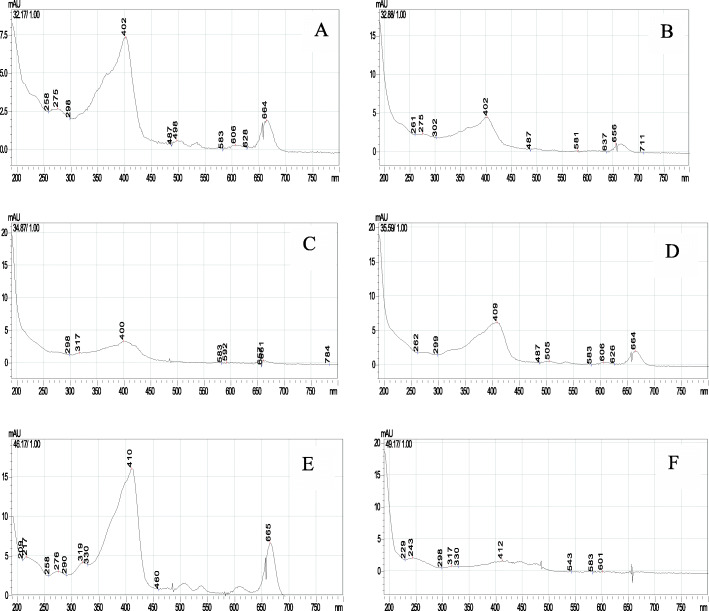
The UV Spectra of FR3T3 sub-fraction peaks using HPLC. Zorbax Eclipse XDB-C18 column, mobile phase acetonitrile – water (9:1 v/v); flowrate 1 mL/min, wavelength 254 nm, **A** Peak with a retention time (Rt) 32.17, **B** Peak with a Rt 32.88, **C** Peak with a Rt 34.87, **D** Peak with Rt 35.59, **E** Peak with Rt 46.17, **F** Peak with Rt 49.17

Spectrum matching was performed from several peaks in FR3T3 to find out more about what compounds in these spectra were likely to be. A spectra peak with retention time of 32.17, 32.88, 34.87, 35.59, and 46.17 min were compatible with the spectra profile of chlorophyll compounds (Fig. 6A-E). Meanwhile, a spectra peak with a 49.17 retention time was unidentified yet (Fig. 6B). Based on TLC profile, the peak was possible to be terpenoids compound (Fig. 5D).

**Fig. 7:**
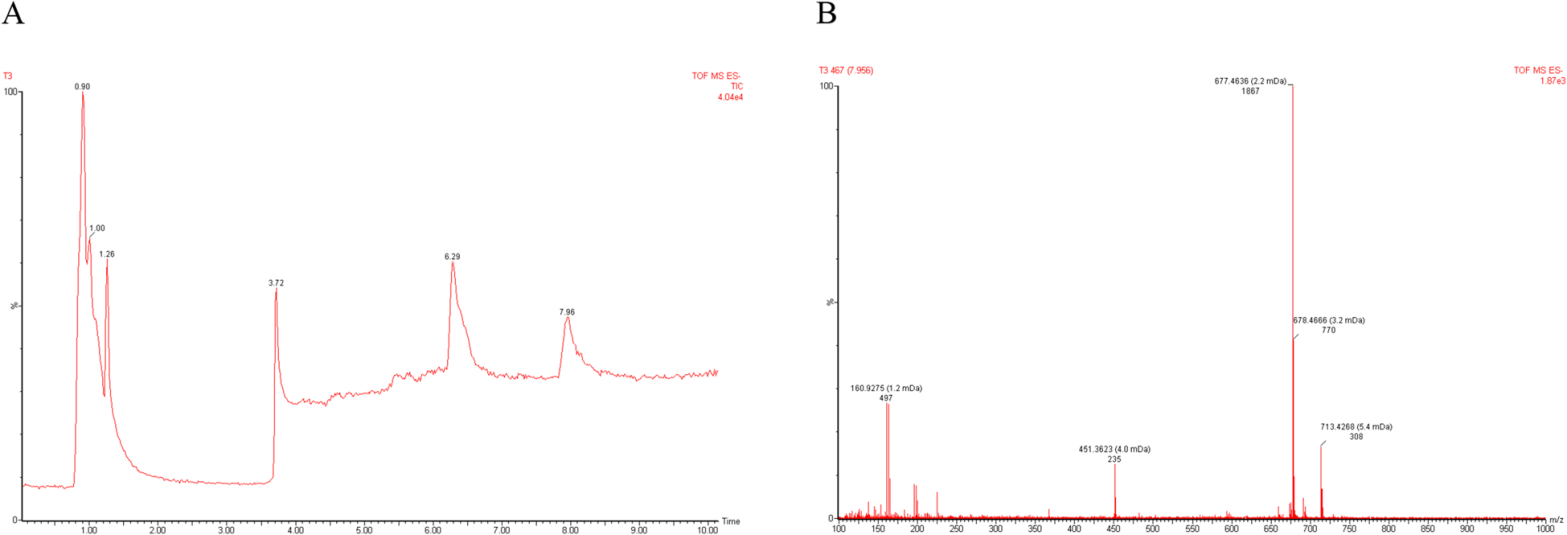
Total ion chromatogram of FR3T3 subfraction (**A**), Mass Spectra of peak with Rt 7.96 min has m/z 677.4636 [M + H] + (**B**)

According to LCMS spectra, the Total Ion Chromatogram (TIC) was detected six peaks. The peak with retention time 0.90; 1.00; 1.26; 3.72; 6.29 and 7.96 have m/z 113.0690; m/z 317.1165; m/z 137.0215; m/z 113.1082; m/z 451.3630 and m/z 677.4636 [M + H]+, respectively (Fig. 7).

### Combining the *A. heterophyllus* dichloromethane extract with current HCV treatments

Next, we compared the IC_50_ of the DCM extract (NaDCM) of *A. heterophyllus* leaves with currently available HCV treatments. The IC_50_ value of NaDCM extract of *A. heterophyllus* was 1.43 μg/mL while Telaprevir, Simeprevir, Ribavirin and Cyclosporin had IC_50_ value of 9.01 nM, 13.09 nM, 10.04 μg/mL, and 0.58 μg/mL respectively (Table 4).

**Table 4:** IC_50_ of *A. heterophyllus* leaves Dichloromethane Extract, Telaprevir, Simaprevir, Ribavirin and Cyclosporin

Sample	IC_**50**_
DCM extract	1.43 ± 0.05 μg/mL
Telaprevir	9.01 ± 0.20 nM
Simeprevir	13.09 ± 1.24 nM
Ribavirin	10.04 ± 0.06 μg/mL
Cyvlosporin A	0.58 ± 0.07 μg/mL

The experiment was performed in triplicate

**Fig. 8:**
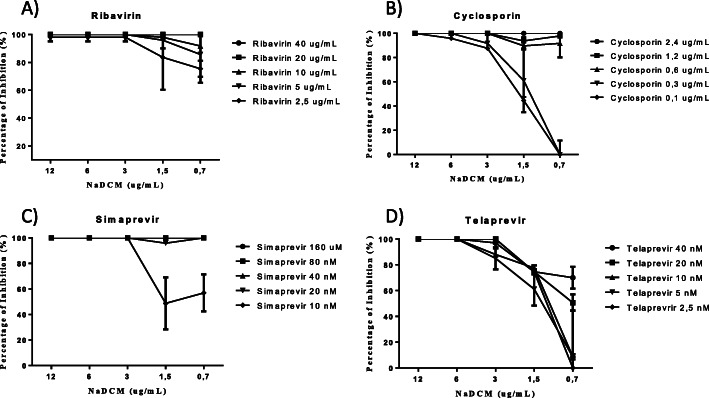
Dose dependence inhibition of **A** Ribavirin, **B** Cyclosporin A, **C** Simaprevir, and **D** Telaprevir against HCV JFH1

We then examined the efficacy of NaDCM as a combination treatment. A 40 and 20 μg/mL Ribavirin treatment combined with NaDCM at all doses examined (0.7–12.0 μg/mL) produced a 100% inhibition of HCV growth. Ribavirin and NaDCM resulted in > 75% inhibition at all combined concentrations (Fig. 8A).

NaDCM and cyclosporin A inhibited 100% of viral growth when Cyclosporin was administered in 2.4, 1.2, and 0.6 μg/mL doses and NaDCM in 12.0, 6.0, and 3.0 μg/mL doses. An inhibition of > 70% of HCV growth was observed when administering ≥0.1 μg/mL dose of Cyclosporin, and ≥ 3 μg/mL of NaDCM (Fig. 8B). When administering ≥20 μM Simeprevir, all concentrations of NaDCM (0.70–12.0 μg/mL) inhibited 100% of HCV growth. The lowest concentrations of NaDCM (0.7 μg/mL) and 10 μM of Simeprevir inhibited 50% of HCV growth (Fig. 8C). Telaprevir inhibited 100% of HCV growth when ≥6 μg/mL NaDCM was administered; however, 1.5 μg/mL of NaDCM lowered the inhibition of all of the telaprevir concentrations tested (Fig. 8D).

**Fig. 9:**
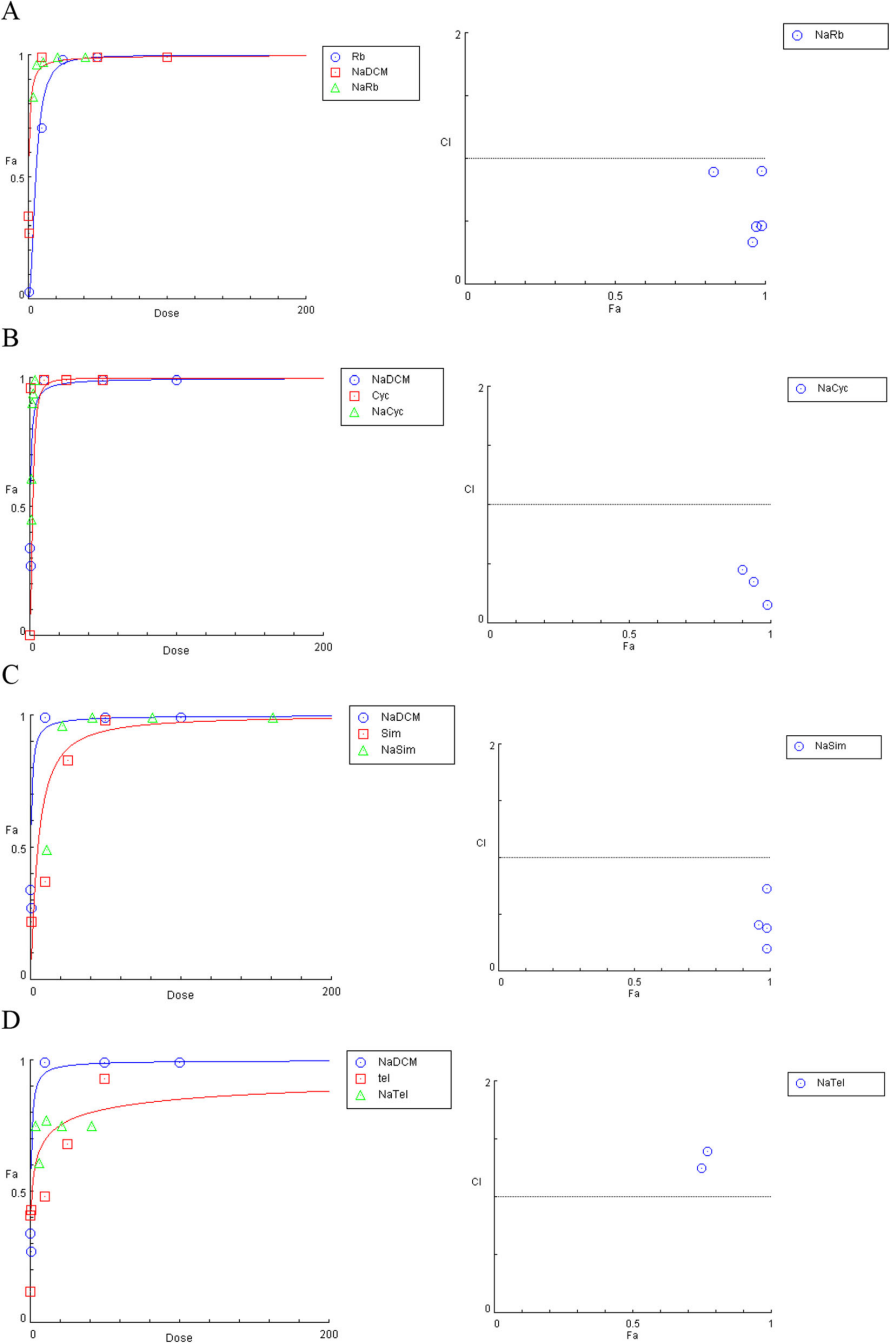
The effect of a 1.5 μg/mL NaDCM dose combined common HCV treatments: **A** Ribavirin, **B** Cyclosporin A, **C** Simaprevir, and **D** Telaprevir

Next, we analyzed the dose-response curves from NaDCM at a concentration of 1.5 μg/mL combined with Ribavirin at 40.0, 20.0, 10.0, 5.0, and 2.5 μg/mL using Compusyn software. The combination index (CI) was < 1, indicating that the two drugs work synergistically (Fig. 9A).

Compusyn analysis also indicated that 0.1, 0.3, 0.6, 1.2, and 2.4 μg/mL doses of Cyclosporin combined with 1.5 μg/mL NaDCM produced CI values of 4.54, 2.37, 0.45, 0.35, and 0.15 respectively. These results suggested that three concentrations produce a synergistic effect while the other two concentrations produce an antagonistic effect. Therefore, a 1.5 μg/mL NaDCM dose should be combined with a minimum dose of 0.6 μg/mL of Cyclosporin for combination therapy (Fig. 9B). All combination doses of Simaprevir except for 10 mM combined with a 1.5 μg/mL dose of NaDCM produced a synergistic effect (CI score < 1; Fig. 9C). All doses of Telaprevir examined combined with a 1.5 μg/mL dose of NaDCM produced CI values that were > 1 indicating antagonism between these two treatments (Fig. 9D). The IC_50_ value of the combination of NaDCM extract (with various concentration of antiHCV drug was showed at Table 5.

**Table 5:** IC_50_ of combination treatment of NaDCM (1.5 μg/mL) with various concentration of antiHCV drug

NaDCM (1.5 μg/mL)					
Ribavirin			Cyclosporin		
Conc (ug/mL)	IC50	combination treatment	Conc (ug/mL)	IC50	combination treatment
40.00	< 0,1	Syn	2.40	< 0,03	Syn
20.00	< 0,1	Syn	1.20	0,03 ± 0,03	Syn
10.00	0.32	Syn	0.60	0,19 ± 0,12	Syn
5.00	0.47	Syn	0.30	1,20 ± 0,60	Ant
2.50	0,4 ± 0,3	Syn	0.10	1,63 ± 0,17	Ant
NaDCM (1.5 μg/mL)					
Simaprevir			Telaprevir		
Conc (nM)	IC50	combination treatment	Conc (nM)	IC50	combination treatment
160.00	< 0,7	Syn	40.00	0,21 ± 0,15	Ant
80.00	< 0,7	Syn	20.00	0,75 ± 0,06	Ant
40.00	< 0,7	Syn	10.00	0,9 ± 0,1	Ant
20.00	< 0,7	Syn	5.00	1,42 ± 0,07	Ant
10.00	0,83 ± 0,33	Ant	2.50	1,22 ± 0,09	Ant

*Syn* Synergism effect, *Ant* Antagonist effect

## Discussion

Many medicinal plants have been reported as promising potential anti-HCV agents, such as *Magnolia officinalis, Maytrenus ilicifolia, Silybum marianum, and Camellia sinensis* [26, 29–31]. Extracts of these plants have been further refined into compounds that have been able to inhibit HCV at various points in its lifecycle. Oleanolic acid and ursolic acid were anti-HCV substances isolated from *Ligustrum lucidum* that could inhibit the HCV NS5B protein [32]. Chalepin and pseudane IX isolated from *Ruta angustifolia* as well as α-mangostin and γ-mangostin isolated from *Gracinia mangostana* were all able to inhibit HCV RNA replication [22, 33]. Saiskoponin b2 isolated from *Bupleurum koil* inhibited viral entry [34].

In a previous study on *A. heterophyllus* leaves as anti-HCV, it was reported that ethanol, methanol, and dichloromethane extracts actively inhibited HCV with IC_50_ values of 12.9 ± 2.6 g/mL, 6.8 ± 0.8 g/mL, and 1.5 ± 0.6 g/mL respectively (Hafid et al., 2017). In this study, the dichloromethane extract was further separated to find the active sub-fraction that played a role in providing anti-HCV activity using bioassay guided isolation. This was the first study to explore the presence of a synergistic effect between a dichloromethane extract of *A. heterophyllus* with several HCV drugs such as Simaprevir, Ribavirin, and Cyclosporin A.

The in vitro assay we performed using the JFH1a strain of HCV and Huh7it-1 cells demonstrated the dichloromethane extract of *A. heterophyllus* sub-fraction FR3T3 possesses anti-HCV properties. This anti-HCV activity occurred mainly through the post-entry stage by reducing NS3 protein expression and RNA replication. Nevertheless, FR3T3 had some anti-HCV activity in the HCV entry stage, demonstrated by the virucidal and cell pretreatment effects we observed; however, it was not as pronounced. FR3T3 was less effective at inhibiting HCV than the dichloromethane extract of *A. heterophyllus*. The dichloromethane of *A. heterophyllus* had an IC_50_ value of 1.43 μg/mL whereas the IC_50_ of the FR3T3 sub-fraction was 4.69 ± 0.95 μg/mL (Table 2). These results suggest the dichloromethane of *A. heterophyllus* is more effective than the sub-fraction we isolated.

Through using Thin-layer Chromatography, we found that FR3T3 contained terpenoid and chlorophyll-related compounds. Some terpenoid compounds have reported as anti-HCV agents such as terpenoids isolated from *Flueggea virosa* [35], triterpenoid saponins from *Platycodon grandiflorum* [36] and diterpen lacton andrographolide from *Andrographis paniculata* [27]. Chlorophyll breakdown compounds from *Morinda citrifolia*, pheophorbide-a and pyropheophorbide-a, have also been identified as anti-HCV substances that inhibit HCV entry and replication [37].

Combination therapy using several drugs that each target different molecular pathways is considered a key strategy to achieve therapeutic success with lower doses. Combining the DCM extract of *A.heterophyllus* concentration 1.5 μg/mL with currently available HCV treatments (Simaprevir, Ribavirin, Cyclosporin A, and Telaprevir) resulted in synergistic effects on Simaprevir, Ribavirin, and Cyclosporin A with CI value < 1. While there is antagonist effect if the active extract (1.5 μg/mL) was used with telaprevir with CI value > 1. Simeprevir is the second generation of HCV NS3/4A and telaprevir is the first generation as HCV NS3/4A protease. Whereas Ribavirin and Cyclosprine act by interfere the host factor [38]. The synergistic effects of these combinations may be useful for patients infected by drug-resistant HCV strains.

Some report have been published about combining natural compound together with several antiviral drugs including as combination treatment for HCV. The combination of several antiviral drugs often show a greater inhibition activity and reduction in HCV RNA level than if it use in single treatment [39]. The curcumin have reported enhances inhibitory effects of boceprevir which known as NS3 protease inhibitor, Cyclosporin A, and Peg-IFN-α [40]. A polyphenol compound, Delphenidin, have improved the effectiveness of both boceprevir and IFN-α [41]. Moreover, the extracts of *Phyllanthus amarus* leaves used in combination with IFN-α exhibit synergistic effect against HCV in Rep 2a cells [42].

## Conclusion

An extract produced from *A. heterophyllus* and its sub-fraction, FR3T3, displayed potential anti-HCV activities in this study. Therefore, they are promising drug, complementary or alternative medicine candidates for HCV infections. FR3T3 mainly inhibited the post-entry stage but produced a slight anti-HCV effect at the entry stage. A combined treatment of the dichloromethane extract of *A. heteropyllus* with Ribavirin, Cyclosporin, and Simaprevir produced synergistic effects.

## Data Availability

The all data used to support the findings of this study are available from the corresponding or the first authors upon request.

## Abbreviations

BSA: Bovine serum albumin
BCA: Bichincronic acid
CC_50_: Cytotoxic concentration 50%
DAAs: Direct acting anti-virals
DMEM: Dulbeco’s Modiffied Eagle Medium
DMSO: Dimethyl sulfoxide
FBS: Fetal Bovine Serum
HCV: Hepatitis C virus
IC_50_: Inhibition concentration 50%
MOI: Multiple of infection
NEAA: Non-essential amino acids
NMR: Nuclear magnetic resonance
PBS: Phosphate Buffer aline
SVR: Sustain virology respond
UV: Ultra violet
VLC: Vacum liquid chromatography
